# Nonlinear Relations Between Resting Heart Rate Measures and Health Risk Behavior in Emerging Adulthood

**DOI:** 10.1002/jad.70175

**Published:** 2026-05-22

**Authors:** Derek P. Spangler, Nina Lauharatanahirun

**Affiliations:** ^1^ Department of Biobehavioral Health Pennsylvania State University University Park Pennsylvania USA; ^2^ Department of Biomedical Engineering Pennsylvania State University University Park Pennsylvania USA

## Abstract

**Introduction:**

Resting heart rate (HR) measures reflect autonomic processes that could predict health risk behavior (HRB) in emerging adulthood when risky behavior is prominent. However, prior studies and extant theories are inconsistent, such that the relationships between HR measures and HRB could be positive or negative in direction. To reconcile these inconsistencies, we predicted that positive and negative relations between HR measures and HRB propensity exist together in a quadratic function.

**Methods:**

The current study tested this nonlinear hypothesis in a sample of young adults (*N* = 89, Mean Age = 21 years, 66% Female) who completed three separate resting baseline periods. ECG was recorded throughout to compute mean HR and high‐frequency heart rate variability (HF‐HRV) as partial and pure indices of resting vagal activity, respectively. HRB propensity was self‐reported on the health/safety and recreational subscales of the Domain‐Specific Risk‐Taking questionnaire.

**Results:**

Resting HR and HF‐HRV each exhibited quadratic associations with HRB propensity that were statistically separate from one another. In all functions, moderate levels of mean HR or HF‐HRV were related to a reduced propensity for risk‐taking behavior.

**Conclusions:**

Findings suggest two potential pathways by which heart rate measures might contribute to HRB in emerging adulthood: one through heightened vagal activity, which may signal under‐arousal, and the other through reduced vagal activity, which may reflect deficient self‐regulation.

## 1 Introduction

Emerging adulthood is a transitional period between adolescence and adulthood (18‐29 years old) that witnesses heightened health risk behavior (HRB), risky actions that cause injury or illness such as reckless driving and substance use (Wood et al. 2018; Willoughby et al. 2021; Scott et al. 2025). HRBs contribute to premature death and later health problems (Keeler and Kaiser 2010; Gibbons and Gerrard 1995; Culatta and Clay‐Warner 2022; Schwartz et al. 2011). Understanding the biological correlates of HRB in young adults is key to building models that predict and prevent risky behavior.

A promising correlate of HRB is the autonomic nervous system (ANS): the neural highway linking the brain and viscera. The ANS is composed of the parasympathetic and sympathetic branches, which regulate homeostasis, stress, and emotion (Jänig 2022; Levenson 2014). Focus has been given to the vagus: a parasympathetic, cardio‐inhibitory nerve with links to adaptive behavior (Thayer and Lane 2009; Porges 2007). Individual differences in vagal activity can be purely indexed with resting high‐frequency heart rate variability (HF‐HRV)—variation in heart rate across the respiratory cycle, 0.15 to 0.4 Hz (Malliani et al. 1991). In contrast, mean heart rate (HR) at rest is predominantly mediated by vagal influences but is also affected by sympathetic action (Cacioppo et al. 1994; Robinson et al. 1966; O'Rourke and Greene 1970). We thus view resting HF‐HRV as a “pure vagal” metric, and resting HR as a “partial vagal” metric that is predominantly (but not solely) determined by parasympathetic influence. These complementary metrics may shed light on the autonomic correlates of health behavior (Alam et al. 2023) and may inform scalable predictors and therapeutic targets for reducing HRB in young adults (Patrick et al. 2016). This capability is limited because prior links between cardiac metrics and HRB are inconsistent.

### 1.1 Inconsistent Links Between HR/HRV and HRB

Lower HR and higher HF‐HRV at rest—which partly signal heightened vagal activity—have both been linked to higher “under‐arousal” and higher cognitive control ability. Under‐arousal and cognitive control show opposing relations to risky behavior. It is thus unsurprising that the direction of cardiac‐HRB relations is inconsistent.

Heightened HRB may arise from *under‐arousal* (Eysenck 1973; Zuckerman 2006; Zuckerman 2007). Under‐aroused individuals are motivated to engage in HRBs since the sensation provided by risky behaviors raise arousal to a level that is rewarding and optimal. Vagal activation decreases cardiovascular arousal, thereby supporting “calm” states (Porges 2007). Higher resting vagal activity may thus reflect the under‐arousal that leads to heightened HRB (Scarpa and Ollendick 2003; Dietrich et al. 2007; Venables 1988; Xu et al. 2014). Indeed, prior studies suggest a *positive association* between vagal metrics and risky behavior where either lower HR or higher HF‐HRV is related to a heightened propensity for risky, impulsive, and/or externalizing behavior (De Pascalis et al. 2007; Hammerton et al. 2018; Sijtsema et al. 2010; Mathias and Stanford 2003; Schmidt et al. 2013; Ortiz and Raine 2004; Lorber 2004; Latvala et al. 2015; Loheide‐Niesmann et al. 2021; Dietrich et al. 2007; Scarpa and Ollendick 2003).

According to the neurovisceral integration model, lower resting vagal activity reflects weaker prefrontal cortex functioning and, as a result, lower cognitive control and self‐regulation capacities (Thayer and Lane 2000, 2009). Lower cognitive control and weaker self‐regulation can lead to unbridled HRB (Ochsner and Gross 2005; Jentsch and Pennington 2014). Contrary to the under‐arousal view, the neurovisceral integration perspective implies a *negative association* where it is lower (not higher) resting vagal activity that is related to heightened risky behavior. Supporting this notion, lower resting HRV is empirically linked to higher risky choice, externalizing behaviors, and polygenic risk for HRB (Forte et al. 2021; Beauchaine 2001; Beauchaine et al. 2007; Beauchaine et al. 2008; El‐Sheikh and Hinnant 2011; Zhang et al. 2017; Xu et al. 2014; Moon et al. 2024; Puhalla et al. 2020; Segarra et al. 2022; Martin et al. 2020).

### 1.2 Quadratic Relations Between Cardiac Metrics and HRB Propensity?

**Figure 1:**
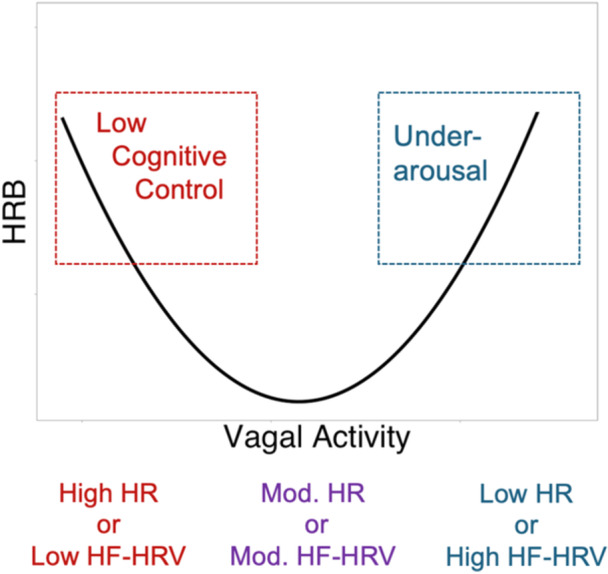
Theorized quadratic associations between cardiac metrics and health risk behavior. *Notes*. HF‐HRV, high‐frequency heart rate variability (pure vagal metric); HR, mean heart rate (partial vagal metric); HRB, health‐risk behavior.

The under‐arousal and neurovisceral integration perspectives suggest opposing relations (positive vs. negative) between resting cardiac metrics and HRB. One potential explanation is that both theories are correct and that the positive and negative cardiac‐HRB relations exist within a quadratic function (Figure 1). A quadratic association is an interaction of a linear effect with itself (HRV*HRV = HRV^2^) where the linear effect changes direction across X (HRV) (Cohen et al. 2013). Resting HRV has been quadratically linked to emotional constructs and cognitive performance, where moderate HRV was associated with the most adaptive outcomes, e.g., the highest positive affect, well‐being, and performance (Kogan et al. 2013, 2014; Spangler et al. 2015, 2021; Duarte and Pinto‐Gouveia 2017; Zhang and Wang 2019; Acland et al. 2019; Mastromatteo et al. 2024). No study has tested nonlinear associations between cardiac metrics and HRB. Such associations could clarify inconsistent theories and identify separate mechanisms of risky behavior. In line with moderate HRV being adaptive, we hypothesize a U‐shaped function (Figure 1) where both low and high HRV are associated with greater HRB engagement relative to moderate HRV. This function may simultaneously capture the opposing effects in the literature that are consistent with the under‐arousal and neurovisceral perspectives. The U‐shaped function is hypothesized to manifest for both resting HR and HF‐HRV; the direction of their *x*‐axes would be reversed relative to one another since the metrics are inversely related.

### 1.3 Current Study

The current study tested quadratic associations between HR metrics and HRB propensity in young adults. We focus on emerging adults given their heightened engagement in problematic risky behavior. Resting HR and resting HF‐HRV served as our “partial vagal" index and “pure vagal" index, respectively. We explored their differential and potentially unique nonlinear relationships with HRBs, since HR and HF‐HRV have shown distinct relations with behavior and health (Alam et al. 2023; Grant et al. 2013). HRB propensity was measured with the health/safety and recreational scales of the Domain‐Specific Risk‐Taking (DOSPERT) questionnaire, a validated self‐report instrument for young adults (Blais and Weber 2006; Shou and Olney 2020). The DOSPERT assesses distinct yet interrelated domains of risk‐taking. Risk‐taking in the health/safety domain (e.g., reckless driving) of the DOSPERT has the clearest mapping onto HRB. However, risk‐taking in the recreational domain (e.g., skydiving) could also be considered health risk behavior since such behaviors can potentially cause serious injury or even death. There is a lack of clarity about these domains’ roles in young adult HRB. We thus explored whether cardiac‐HRB relations differed between health/safety and recreational domains. We hypothesized U‐shaped quadratic associations (Figure 1) between each cardiac metric (HR, HF‐HRV) and each risk‐taking metric (health/safety, recreational). Differences in effects between metrics were tested in an exploratory fashion.

## 2 Methods

### 2.1 Participants

Participants (*N* = 120) were a part of larger study on the cardio‐behavioral correlates of threat responding and decision‐making. They were adults with good overall physical health. The exclusionary criteria were: (1) a current or prior diagnosis of a cardiovascular, metabolic, or neurological condition, (2) nicotine use, and (3) diagnosis of COVID‐19 in the past 2 weeks. Participants were either college students or community members in the area around Penn State who were recruited through flyers, social media, word‐of‐mouth, and an online platform (StudyFinder). Although not of primary focus to the current paper, participants were randomly assigned to complete study procedures during either the day or night. Time‐of‐day group was controlled for in the statistical analyses. To mitigate noise in the physiological signals, participants abstained from: (1) alcohol for 24 h, (2) caffeine for 6 h, (3) eating for 2 h, and (4) vigorous exercise for 2 h. Abstention criteria were validated with a questionnaire at the start of the study session. Twenty‐three participants were removed from analyses because of errors in the equipment used for data collection, thereby leaving 97 participants. Since the current paper focuses on young adults, we additionally excluded eight participants who had an age of 30 years or older. Our final sample consisted of 89 individuals between the ages of 18 and 29 years, a range that is often used to characterize young adulthood (Arnett et al. 2014). The final sample (*N* = 89) was mostly female (66.3%) and Caucasian (69.7%) with a mean age of 21.2 years (SD = 2.9). All procedures were approved by the University institutional review board were carried out in accord with the Declaration of Helsinki. All participants provided informed consent before study procedures.

### 2.2 Procedure

Procedures were carried out by two trained experimenters in a well‐lit, temperature‐controlled room. After participants ensured abstention criteria and provided informed consent, they were attached to the physiological recording hardware via electrodes and lead wires. Participants then completed questionnaires, including the DOSPERT scales, on a computer. After the questionnaires, participants completed an image‐decision task involving a series of lottery choices that were each preceded by an image. Images varied in the emotional content (threat of injury, threat of infection, neutral). The trials were chunked into three separate blocks composed of 60 trials each. Each block was preceded by a 2‐min resting baseline where participants remained seated. This yielded three resting baselines periods for each participant. To ensure a proper resting state, participants sat in front of a computer and were instructed to look at a black screen while remaining as still and quiet as possible. Participants wore headphones throughout the session to mitigate distraction. After the image‐decision task and baselines, participants were given $25 in compensation, and bonus compensation (maximum of $5) was also provided based on a randomly selected decision trial. The total duration of the session was ~90 min.

### 2.3 Baseline ECG Recordings

Cardiac measures were derived from surface ECG recordings. The ECG was measured at a modified Lead II configuration with adhesive spot electrodes on the skin at the lower left ribcage (+) and below the clavicles (−, GRD). The signal was routed to the BIOPAC ECG 100C where the signal was amplified and filtered and then digitized by the BIOPAC MP160 (BIOPAC Systems Inc., Goleta, CA, USA). These digitized data were recorded on a PC using AcqKnowledge software (BIOPAC Systems Inc., Goleta, CA, USA); data were saved for offline analysis. ECG signals were bandpass filtered (0.5–35 Hz). The filtered signal was submitted to a modified Pan‐Tompkins algorithm to detect successive R‐spikes, leading to the construction of the R‐R interval time courses. Artifact correction was implemented in two steps where (i) misclassified R‐spikes were first corrected in AcqKnowledge by trained research assistants and (ii) abnormal RR intervals (< 300 ms or > 2000 ms; or more than 30% different than the prior RR interval) were replaced with cubic spline interpolation in RStudio. Abnormal RR intervals constituted < 1% of data. Baseline periods that had more than three consecutive abnormal intervals were excluded from the statistical analyses, and HR/HRV metrics were imputed for these periods (described in Data Preparation and Statistical Analysis). Thoracic impedance was also collected using four spot electrodes on the back and posterior neck, which provided an estimate of respiration rate for follow‐up analyses (see Supplemental Materials S1).

### 2.4 Measures

#### 2.4.1 HRB Propensity

Propensity for engaging in HRB was self‐reported by participants on two subscales from the Revised Domain‐Specific Risk‐Taking (DOSPERT) (Blais and Weber 2006). The Health/Safety and Recreational subscales were employed. Each subscale had six items where every item listed a different behavior or activity (e.g., Health/Safety: ‘Driving a car without wearing a seat belt’; Recreational: ‘Going down a ski run that is, beyond your ability’). Participants rated each item based on their likelihood of engaging in that behavior/activity. Ratings were made on a 7‐point Likert scale ranging from 1 = ‘*Extremely unlikely*’ to 7 = ‘Extremely Likely.’ Item‐level scores were averaged for each subscale to yield separate total scores in the health/safety and recreational domains. Higher scores on each subscale indicated a stronger propensity for risk‐taking or engaging in HRB. The internal consistency of the recreational subscale was high, *Cronbach's alpha* = 0.83, suggesting good reliability. In contrast, the health/safety subscale demonstrated lower consistency, *Cronbach's alpha* = 0.47. See Supplemental Materials S1 (‘Parsing the Health‐Safety DOSPERT Subscale’) for follow‐up item‐level correlations and follow‐up analyses split by different items of the health/safety subscale.

#### 2.4.2 HR Metrics

HR metrics were computed separately for each resting baseline using RStudio. Mean HR in beats per minute (bpm) was calculated as the average of HR values in each baseline (60,000/R‐R intervals [ms]). Mean HR served as our partial vagal metric. High‐frequency heart rate variability (HF‐HRV) was calculated as mean spectral power (ms^2^) in the 0.15–0.4 Hz band, derived from a short‐time Fourier transform on the resampled (4 Hz via cubic spline interpolation) R‐R intervals. Power was computed in 60 s windows with 30 s (50%) overlap. HF‐HRV served as our pure vagal metric. HF‐HRV values were computed using the RHRV package in RStudio (Martínez et al. 2017). HF‐HRV scores were natural logarithm (ln) transformed to reduce their positive skew; the transformed variables are denoted as “lnHF‐HRV” in subsequent sections. Mean HR scores from the three baselines were averaged to arrive at a single resting HR scores per person. The three baseline mean HR scores were strongly correlated with one another, *r*s > 0.90, *p*s < 0.05. Similarly, lnHF‐HRV scores from each baseline were also averaged to index resting ln HF‐HRV; the three baseline lnHF‐HRV scores were also strongly correlated with one another, *r*s > 0.78, *p*s < 0.05. Our primary results focus on these averaged HR and lnHF‐HRV scores. Such averaging aimed to provide more reliable, trait‐like measures compared to focusing on single baseline scores. Results split by baseline are detailed in the Supplemental Materials S1.

### 2.5 Data Preparation and Statistical Analysis

Approximately 1% of the item‐level responses were missing from the DOSPERT scales. Due to too many artifactual RR intervals (see above), two participants were missing mean HR and lnHF‐HRV scores for one resting baseline, both of which being the third resting baseline. We imputed the missing scores using multiple imputation with the *mice* package in R (Van Buuren 2007; Zhang 2016). The final imputed scores were computed as the mean across five separate imputations. Imputation helps avoid bias that would be caused by instead listwise deleting participants with missing data (Van Ginkel et al. 2020). Data were next screened for outliers using Tukey's (1977) Rule, where values were defined as outliers if they were less than the lower fence (25th percentile − 1.5*interquartile range) or higher than the upper fence (75th percentile + 1.5*interquartile range). Outliers were Winsorized by replacing the outlier value with the nearest fence value. The procedure only affected resting HR scores where one case was Winsorized for each resting HR variable.

Quadratic relations between the cardiac and DOSPERT metrics were tested using hierarchical, robust linear regression. DOSPERT risk‐taking scores served as the outcome measure, and the cardiac metrics served as the covariates. Linear and quadratic terms for resting HR and resting lnHF‐HRV were represented by grand‐mean centering each resting measure (e.g., HR) and then squaring this centered metric (e.g., HR*HR). As noted earlier, resting HRV and lnHF‐HRV were represented as the average scores across the three baselines. To explore differential relationships, models were conducted separately for each DOSPERT subscale (health/safety *vs.* recreational) and cardiac measure (HR vs. lnHF‐HRV). We also sought to ensure that statistically significant relations were not driven by confounds. Therefore, anytime significant relations were detected, we next entered in covariates to test whether such effects survived control for confounds that are feasibly related to HR/HRV or risk‐taking. The initial covariates were: (1) Gender (0 = *female*, 1 = *male*), (2) Body mass index (BMI) (kg/m^2^), and (3) time‐of‐day condition (0 = *day*, 1 = *night*). We also sought to understand whether significant effects involving HF‐HRV were driven by mean HR or vice versa. We thus additionally entered the other cardiac metric into the regression model after initially controlling for gender, BMI, and time‐of‐day. This hierarchical approach allowed us to isolate whether it was the initial covariates versus the other cardiac metric that was driving any significant cardiac‐HRB relationship. Nine participants reported having asthma. Follow‐up regressions were conducted without these individuals, and the results remained highly similar (*p* < 0.05, not presented). We therefore presented results from the full models with asthmatic participants (*N* = 89). Lastly, given concerns that respiration rate might confound HF‐HRV (Grossman et al. 1991), we performed a sensitivity analysis that additionally adjusted for respiration rate. The focal effects for lnHF‐HRV and HR remained similar and are detailed in the Supplemental Materials S1. For all models, regression assumptions were carefully screened by inspecting the residuals, and outlier residual values were observed. To mitigate the influence of outliers, robust regression using an M‐M type estimator was carried out for all models. All analyses were implemented in RStudio using base *R* and the “robustbase” package (Maechler et al. 2021). All *p*‐values were two‐tailed and compared to an alpha of 0.05.

## 3 Results

Table 1 includes descriptive statistics. The health/safety and recreational DOSPERT scores were positively and significantly correlated, *r* = 0.35, *p* = 0.0007. As expected, the average resting HR and average resting lnHF‐HRV metrics were negatively and significantly correlated, *r* = −0.53, *p* < 0.0001.

**Table 1:** Descriptive statistics (*N* = 89).

	Mean (or %)	SD	Median	Min–Max
Age	21.19	2.93	20	18–29
Gender (% female)	66.29%	—	—	—
Racial identity (% Caucasian)	69.66%	—	—	—
Resting HR—first baseline (bpm)	75.53	10.68	74.47	51.92–106.84
Resting HR—second baseline (bpm)	77.18	10.83	76.50	56.17–106.27
Resting HR—third baseline (bpm)	77.25	10.80	75.31	55.59–109.15
Average resting HR (bpm)	76.66	10.56	75.69	54.56–108.53
Resting lnHF‐HRV—first baseline (ln[ms^2^])	6.13	1.12	6.23	3.29–8.23
Resting lnHF‐HRV—second baseline (ln[ms^2^])	5.84	1.09	5.75	3.15–8.16
Resting lnHF‐HRV—third baseline (ln[ms^2^])	5.99	1.09	5.92	3.63–8.57
Average resting lnHF‐HRV (ln[ms^2^])	5.99	1.04	5.92	3.50–8.22
DOSPERT—Health/Safety	2.83	0.96	2.83	1–4.83
DOSPERT—Recreational	3.68	1.58	3.67	1–6.67
DOSPERT—Total	3.25	1.06	3.33	1.08–5.58

Abbreviations: DOSPERT, domain‐specific risk‐taking scale; HR, mean heart rate; lnHF‐HRV, natural log transformed high‐frequency heart rate variability (0.15–0.40 Hz).

### 3.1 Resting HR is Quadratically Related to Health/Safety Risk‐Taking

**Figure 2:**
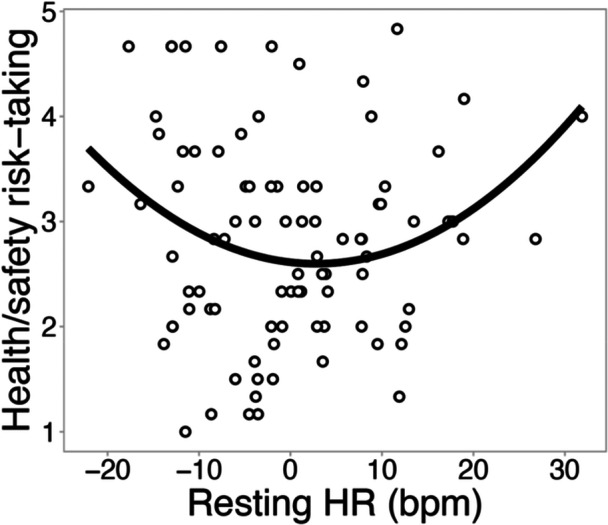
Quadratic association between resting heart rate and health/safety risk‐taking propensity (DOSPERT). *Notes*: Resting HR = mean heart rate, averaged across the three resting baselines. HR scores are in beats per minute (bpm) units and are grand‐mean centered.

**Health/Safety.** We first tested resting HR's linear and quadratic associations with risk‐taking propensity in the health/safety domain. The linear term for HR was not statistically significant, *β* = −0.11, *SE* = 0.11, *p* = 0.345, *95% CI* [−0.34, 0.12]. However, the quadratic term was significant, *β* = 0.29, *SE* = 0.08, *p* = 0.0007, *95% CI* [0.13, 0.45], indicating a *U*‐shaped association between resting HR and health/safety risk‐taking. Relative to moderate HR, both low and high levels of resting HR were related to a higher propensity for risky behavior in the health/safety domain (Figure 2). The quadratic association between HR and health/safety risk‐taking remained statistically significant and comparable in size when statistically adjusting for gender, BMI, and time‐of‐day, *β* = 0.30, *SE* = 0.08, *p* = 0.0004, *95% CI* [0.13, 0.46], and when additionally controlling for lnHF‐HRV's linear and quadratic terms, *β* = 0.24, *SE* = 0.09, *p* = 0.010, *95% CI* [0.06, 0.42]. As indicated in the Supplemental Materials S1, the nonlinear relation between HR and health/safety risk‐taking was detected when using all items from this subscale.

**Recreational.** We next tested resting HR's associations with risk‐taking propensity in the recreational domain. Both the linear, *β* = ‐0.06, *SE* = 0.12, *p* = 0.603, *95% CI* [−0.29, 0.17] and quadratic terms, *β* = 0.05, *SE* = 0.10, *p* = 0.588, *95% CI* [−0.14, 0.25] for HR were not statistically significant. Both effects remained non‐significant when adjusting for covariates (*p* > 0.05, results not presented).

### 3.2 Resting lnHF‐HRV is Quadratically Related to Both Health/Safety and Recreational Risk‐Taking

**Figure 3:**
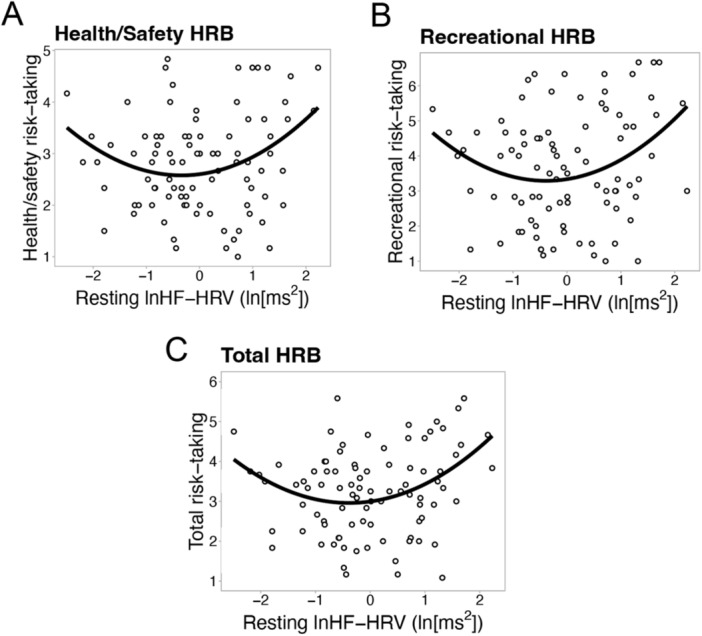
Quadratic associations between resting heart rate and risk‐taking propensity (DOSPERT). Separate effects are presented for the different risk‐taking measures: (A) Health/Safety, (B) Recreational, (C) Total = average of health/safety and recreational risk‐taking subscales. *Notes*: Resting lnHF‐HRV = high‐frequency heart rate variability, averaged across the three resting baselines. LnHF‐HRV scores represent mean spectral power of R–R intervals in the 0.15–0.40 Hz band. Scores are in natural logarithm transformed milliseconds‐squared (ln[ms^2^]) units and are grand‐mean centered.

**Health/Safety.** As with resting HR above, resting lnHF‐HRV's linear and quadratic associations were tested in relation to risk‐taking propensity in the health/safety domain. Unlike the linear term, *β* = 0.14, *SE* = 0.10, *p* = 0.159, *95% CI* [−0.06, 0.35], the quadratic term for lnHF‐HRV was statistically significant, *β* = 0.27, *SE* = 0.08, *p* = 0.002, *95% CI* [0.10, 0.43]. The quadratic association mirrors the analogous U‐shaped quadratic effect involving resting HR; that is, moderate lnHF‐HRV was associated with the lowest risk‐taking (Figure 3A). The quadratic effect between lnHF‐HRV and health/safety risk‐taking remained statistically significant and similar in size when adjusting for gender, BMI, and time‐of‐day, *β* = 0.30, *SE* = 0.10, *p* = 0.003, *95% CI* [0.11, 0.49], and when additionally adjusting for resting HR's linear and quadratic terms, *β* = 0.24, *SE* = 0.10, *p* = 0.017, *95% CI* [0.04, 0.44]. LnHF‐HRV's quadratic association with health/safety risk‐taking was driven by risk‐taking items that assessed pleasure‐seeking behaviors (risky sex and binge drinking). See the Supplemental Materials S1 for more details.

**Recreational.** When estimating risk‐taking in the recreational domain, resting lnHF‐HRV did not exhibit a significant linear term, *β* = 0.16, *SE* = 0.11, *p* = 0.145, *95% CI* [−0.06, 0.37], but it did exhibit a significant quadratic term, *β* = 0.25, *SE* = 0.10, *p* = 0.010, *95% CI* [0.06, 0.45]. That U‐shaped quadratic association is depicted in Figure 3B and mirrors the quadratic association between lnHF‐HRV and health/safety risk‐taking reported above. The quadratic effect remained significant and similar in size when adjusting for gender, BMI, and time‐of‐day, *β* = 0.27, *SE* = 0.10, *p* = 0.008, *95% CI* [0.07, 0.47], and when additionally adjusting for HR's linear and quadratic terms, *β* = 0.27, *SE* = 0.10, *p* = 0.010, *95% CI* [0.07, 0.48].

**Combined Across Risk Domains.** Overall, there were similar nonlinear effects of lnHF‐HRV across health/safety and recreational risk‐taking domains. We therefore re‐ran the regression model such that the dependent measure (risk‐taking) averaged across scales to reflect a general HRB propensity. The model revealed a non‐significant linear term, *β* = 0.19, *SE* = 0.10, *p* = 0.058, *95% CI* [−0.01, 0.38], but a significant quadratic term, *β* = 0.30, *SE* = 0.08, *p* = 0.0006, *95% CI* [0.13, 0.47]. Mirroring the individual effects above (Figure 3A,B), this *U*‐shaped quadratic effect is plotted in Figure 3C.

## 4 Discussion

We found evidence that resting HR and HF‐HRV are both quadratically associated with HRB propensity in young adults. Within these nonlinear associations, young adults with moderate levels of resting HR or lnHF‐HRV reported the lowest HRB propensity relative to their counterparts with lower or higher values on the same cardiac metrics. These associations appear to be robust since they survived covariate adjustment. Findings were generally aligned with our predictions in that they mostly emerged across cardiac metrics and HRB domains (health/safety and recreational). Resting HR (partial vagal index) was nonlinearly related to risk‐taking in the health/safety domain, a prototypical HRB context involving binge drinking, failure to wear a safety belt, etc., but was unrelated to risk‐taking in the recreational domain. In contrast, resting HF‐HRV (pure vagal index) exhibited nonlinear associations with HRB both in the health/safety and recreational domains. Young adults’ resting HF‐HRV may thus signal a more general tendency to engage in potentially harmful behaviors even in recreational contexts, an understudied HRB domain. The nonlinear effects of HR and HF‐HRV were also statistically separate from one another, possibly suggesting their unique roles in unhealthy risk‐taking. Broadly, the nonlinear relations reconcile inconsistent positive and negative associations between HR/HRV and risk‐taking metrics (e.g., De Pascalis et al. 2007; Hammerton et al. 2018; El‐Sheikh and Hinnant 2011; Zhang et al. 2017). We show that both linear relations exist in a larger quadratic association that may be missed without testing curvilinear terms. The present study thus adds to a growing literature on curvilinear relations involving HR/HRV while also highlighting novel quadratic relations with risk‐taking specifically (e.g., Kogan et al. 2013, 2014; Spangler et al. 2021; Acland et al. 2019).

### 4.1 Theoretical Importance of Nonlinear Effects

The aspects of resting HR and HF‐HRV that drive HRB possibly reflect vagal activity. A dominant vagal basis for resting HR is credible; prior autonomic blockade studies suggest that resting HR is mostly determined by vagal activity, while HF‐HRV is solely determined by vagal activity (Cacioppo et al. 1994; Robinson et al. 1966; Pomeranz et al. 1985). We hence refer to resting HR and HF‐HRV as “vagal metrics,” while recognizing possible sympathetic influences on resting HR.

The present quadratic effects may suggest dual theoretical mechanisms that drive vagal activation and its links to risk‐taking. The prior positive linear relations between vagal and risk‐taking metrics have been explained by under‐arousal theory (Eysenck 1973; Zuckerman 2006; Scarpa and Ollendick 2003; Dietrich et al. 2007; Venables 1988). The negative links between vagal and risk‐taking metrics have been explained by self‐regulation in the neurovisceral integration model (Thayer and Lane 2009). Our nonlinear associations, which encompass both negative and positive linear relations, may suggest that HRB is driven by *both* greater under‐arousal and weaker self‐regulation. With those mechanisms in mind, young adults with lower resting HR at the left side of the function (Figure 2) may have a higher HRB propensity because they are under‐aroused at baseline and require the stimulation of risk‐taking to raise arousal to an optimal level. Young adults with higher resting HR at the right side may have a higher HRB propensity because they lacked the cognitive control capacities to properly inhibit risk‐taking behaviors that might harm their health. The same interpretations can be used for the HRV effects but flipped along the *x*‐axis (Figure 3). The speculated mechanisms are plausible considering their importance in the dual system framework, a well‐established model of risk‐taking (Steinberg 2010). The framework underscores both high approach motivation and low cognitive control as factors that drive HRB in young adults and adolescence. Under‐arousal is a form of approach motivation because under‐arousal is uncomfortable, galvanizing impulsive actions that are arousal‐inducing and rewarding (Eysenck 1973; Zuckerman 2007). Irrespective of specific explanations, the current quadratic effects qualify the neurovisceral integration model's (Thayer and Lane 2009) assertion that high vagal activity is the most adaptive for behavior.

### 4.2 Differential Effects of HR and HRV

By showing that HR and HF‐HRV explain unique variance in HRB, current findings add to the growing notion that mean HR and HRV are distinct but complementary constructs in the estimation of human behavior (Panina et al. 1995; Grant et al. 2013; Alam et al. 2023) *If HR and HRV are distinct, then what do they represent?* Resting HF‐HRV is an established index of respiratory‐linked vagal modulation (Malik and Camm 1993; Yasuma and Hayano 2004). Resting mean HR has been proposed to better index vagal tone, such that mean HR reflects tonic vagal activation across inspiration and expiration (Grossman and Kollai 1993). However, sympathetic and parasympathetic branches can dually influence mean HR (Cacioppo et al. 1994). Low HR in the current function (Figure 2) might therefore reflect diminished sympathetic activity alongside heightened vagal activity, which jointly contribute to under‐arousal; conversely, high HR may reflect heightened sympathetic activity and reduced vagal activity that jointly support self‐regulation. While neurophysiological distinctions can be made, it is unclear how such distinctions separately give rise to HRB. At best, our results suggest that both mean HR and HRV play roles in risk‐taking, with a need for future work to pinpoint the psychophysiological mechanisms that underlie differential cardiac‐HRB relationships.

*Resting HF‐HRV* = *a general correlate of HRB propensity?* Further highlighting their distinction, HR and HF‐HRV's pattern of relations with the HRB domains slightly differed. Both HR and HF‐HRV exhibited nonlinear associations with risk‐taking in the health/safety domain, reflecting prototypical HRBs such as substance use. Unlike resting HR, resting HF‐HRV was also related to HRB in the recreational context. It should be noted that recreational risk‐taking could sometimes be adaptive because it often involves physical exercise, is related to higher intrinsic motivation, self‐efficacy, and other “healthy” activities (Lee and Blais 2014; Hernández‐Méndez et al. 2024; Tyne et al. 2024). However, many of the risk‐taking behaviors in the DOSPERT scale, for example, skydiving, carry serious risk of injury and even death. The fact that resting HF‐HRV (but not HR) was related to both risk‐taking domains suggests that vagal modulation may better tap into a general risk‐taking tendency. Indeed, the broader relationships between HF‐HRV and risk‐taking in both HRB domains is consistent with the neurovisceral integration model where resting HRV taps into emotional/cognitive “traits” that persist across varied situational domains (Thayer and Lane 2009). The general mechanism reflected in HF‐HRV could involve sensation‐seeking. This inference is plausible since (1) HF‐HRV's nonlinear relation with health/safety risk‐taking was driven by pleasure‐seeking items (see Supplemental Material S1), and (2) most items in the recreational subscale tap into high‐sensation experiences such as skydiving.

### 4.3 Limitations and Future Directions

The study has several limitations that should be addressed by future work. First, we focused on propensity for engaging in HRB and did not measure risk‐taking behaviors directly. We only used two scales from the (DOSPERT), limiting our ability to assess all the domains of risk‐taking. It is therefore unclear whether HRV truly correlates with a general risk‐taking tendency. Future studies might use task paradigms or ecological momentary assessment to better measure risk‐taking across domains. Second, we did not measure the specific under‐arousal and cognitive mechanisms that possibly explain how low and high vagal activity are related to heightened HRB propensity. Future work should leverage self‐report and task measurement of arousal and cognitive control. Third, some researchers may label our HF‐HRV metrics as “ultra‐short‐term” (< 5 min) and question their reliability (Shaffer et al. 2020). Research is beginning to establish the acceptable reliability and validity of ultra‐short‐term HRV metrics less than 2 min relative to short‐term recordings (e.g., 5 min) (Burma et al. 2021; Baek et al. 2015). The resting HF‐HRV metrics in the current study demonstrated acceptable reliability since they: (i) were highly intercorrelated with one another, and (ii) exhibited similar nonlinear associations with HRB propensity for each baseline period (see Supplemental Material S1), and when averaging across all three baseline periods (Results section). Fourth, future work should additionally include sympathetic measures to test sympathetic contributions to under‐arousal/cognition; this might clarify mean HR's association with HRB. Fifth, prior work has shown that nonlinear relations involving HRV are limited to women (Spangler et al. 2021). The current study was underpowered to examine gender differences, but a female‐specific effect is possible since our sample was primarily composed of women.

### 4.4 Implications

Emerging adulthood represents a critical developmental window where youth exhibit shifts toward greater independence and autonomy leading to increased opportunities for engagement in HRBs. This includes texting while driving, failing to wear a safety belt, binge drinking, and cannabis use. Young adulthood is probably not a monolith: there are individual differences in risk‐taking within this developmental period (Caspi et al. 1997). To that end, our results imply two distinct autonomic endophenotypes that may give rise to individual differences in unhealthy risk taking. This information may aid early identification efforts in young adults, whereby HR measures can offer clues about which individuals are prone to making dangerous choices. Young adults with different HR‐related phenotypes may also benefit from different interventions, which is a topic that should be further explored in future studies. Use of cardiac measures for such early identification/intervention efforts is both practical and scalable across varied clinical and research domains (Nelson et al. 2020; Natarajan et al. 2020).

## Author Contributions

**Derek P. Spangler:** conceptualization, investigation, methodology, validation, visualization, writing – review and editing, formal analysis, project administration, writing – original draft. **Nina Lauharatanahirun:** conceptualization, investigation, methodology, writing – review and editing, project administration.

## Ethics Statement

All procedures were approved by the institutional review board at Penn State and were carried out in accord with the Declaration of Helsinki.

## Consent

All participants provided informed consent before study procedures.

## Conflicts of Interest

The authors declare no conflicts of interest.

## Supporting information

Supporting File

## Data Availability

The data that support the findings of this study are available from the corresponding author upon reasonable request.
