# Supplementary material for: Nonlinear Relations Between Resting Heart Rate Measures and Health Risk Behavior in Emerging Adulthood
Source: J Adolesc. 2026 May 22;98(5):1705–14. doi: 10.1002/jad.70175 (PMC13338657; doi:10.1002/jad.70175)
Supplement: Supplementary file 1 — Supporting File [file JAD-98-1705-s001.docx]

**Nonlinear Relations by Baseline Period**

**Mean HR and Health/Safety Risk-Taking**

**First baseline.** At the first (“pure”) baseline, the linear term for mean HR was not significantly related to health/safety risk-taking propensity, *β* = -0.12, *SE* = 0.11, *p* = 0.278, *95% CI* [-0.34, 0.10]. However, the quadratic term representing the nonlinear relationships between HR and health/safety risk-taking was significant, *β* = 0.33, *SE* = 0.08, *p* = 0.0001, *95% CI* [0.16, 0.49]. See Figure S1A. The function exhibited a U-shape and suggests that relative to lower and higher HR scores, moderate HR was related the lowest propensity for health/safety risk-taking.

**Second baseline**. At the second baseline, the pattern of statistical relationships was identical to those detected using the first baseline HR scores above. Unlike the linear term, *β* = -0.09, *SE* = 0.11, *p* = 0.444, *95% CI* [-0.32, 0.14], the quadratic term was statistically significant, *β* = 0.24, *SE* = 0.09, *p* = 0.008, *95% CI* [0.07, 0.42]. That quadratic relation is depicted in Figure S1B and suggests a U-shaped relation between mean HR and risk-taking propensity in the health/safety domain.

**Third baseline.** The pattern of effects for mean HR at the third baseline were the same as those for the first and second baseline periods. The linear term was not statistically significant, *β* = -0.09, *SE* = 0.12, *p* = 0.447, *95% CI* [-0.34, 0.15], but the quadratic term for mean HR was significant, *β* = 0.26, *SE* = 0.09, *p* = 0.004, *95% CI* [0.09, 0.44]. As indicated in Figure S1C, there was a U-shaped quadratic relationship between mean HR at the third baseline and health/safety risk-taking propensity.

**Summary.** At each of the three baseline periods, mean HR exhibited a U-shaped quadratic association with risk-taking propensity in the health/safety domain.

**Mean HR and Recreational Risk-Taking**

**First baseline.** Mean HR at the first baseline did not exhibit a significant linear, *β* = -0.04, *SE* = 0.11, *p* = 0.750, *95% CI* [-0.26, 0.19], or quadratic term, *β* = 0.03, *SE* = 0.10, *p* = 0.740, *95% CI* [-0.17, 0.24], in the regression model estimating recreational risk-taking propensity.

**Second baseline.** As above, both the linear, *β* = -0.09, *SE* = 0.12, *p* = 0.431, *95% CI* [-0.32, 0.14], and quadratic terms, *β* = 0.09, *SE* = 0.10, *p* = 0.350, *95% CI* [-0.11, 0.31], for mean HR at the second baseline were not statistically significant.

**Third baseline.** Replicating the results above, the linear, *β* = -0.04, *SE* = 0.11, *p* = 0.706, *95% CI* [-0.27, 0.18], and quadratic, *β* = 0.03, *SE* = 0.09, *p* = 0.773, *95% CI* [-0.16, 0.21], terms for HR at the third baseline were not statistically significant.

**Summary.** For all three baseline periods, mean HR did not exhibit a linear or quadratic relationship with risk-taking propensity in the recreational domain.

**LnHF-HRV and Health/Safety Risk-Taking**

**First baseline.** At the first (“pure”) baseline, the linear term for lnHF-HRV was not significantly related to health/safety risk-taking propensity, *β* = 0.17, *SE* = 0.11, *p* = 0.112, *95% CI* [-0.04, 0.38]. Nevertheless, the quadratic term was significant, *β* = 0.25, *SE* = 0.10, *p* = 0.009, *95% CI* [0.07, 0.44]. See Figure S2A for the U-shaped quadratic association between lnHF-HRV and health/safety risk-taking.

**Second baseline.** Unlike the linear term, *β* = 0.09, *SE* = 0.10, *p* = 0.352, *95% CI* [-0.11, 0.30], the quadratic term for lnHF-HRV at the second baseline period was statistically significant, *β* = 0.24, *SE* = 0.07, *p* = 0.002, *95% CI* [0.10, 0.39]. As indicated in Figure S2B, there was a U-shaped quadratic relation that was similar to the nonlinear relation above using first-baseline lnHF-HRV.

**Third baseline.** Replicating the prior baseline effects, the quadratic term, *β* = 0.23, *SE* = 0.09, *p* = 0.010, *95% CI* [0.06, 0.40], but not the linear term, *β* = 0.18, *SE* = 0.11, *p* = 0.106, *95% CI* [-0.04, 0.40], for third-baseline lnHF-HRV was statistically significant. This significant U-shaped quadratic relation between lnHF-HRV and health/safety risk-taking is depicted in Figure S2C.

**Summary.** At each of the three baseline periods, lnHF-HRV exhibited a U-shaped quadratic association with risk-taking propensity in the health/safety domain.

**LnHF-HRV and Recreational Risk-Taking**

**First baseline.** LnHF-HRV at the first baseline period exhibited a quadratic association with recreational risk-taking propensity, *β* = 0.22, *SE* = 0.10, *p* = 0.034, *95% CI* [0.02, 0.42]. The linear term was not significant, *β* = 0.19, *SE* = 0.12, *p* = 0.107, *95% CI* [-0.04, 0.43]. As shown in Figure S3A, this quadratic association took a U-shape like the associations detected for lnHF-HRV and health/safety risk-taking.

**Second baseline.** Mirroring the first baseline, second-baseline lnHF-HRV had a significant quadratic term, *β* = 0.26, *SE* = 0.09, *p* = 0.003, *95% CI* [0.09, 0.43], but a non-significant linear term, *β* = 0.19, *SE* = 0.10, *p* = 0.072, *95% CI* [-0.02, 0.39]. Those results indicate another U-shaped quadratic association (Figure S3B).

**Third baseline.** The linear term for lnHF-HRV at the third baseline was not statistically significant, *β* = 0.09, *SE* = 0.12, *p* = 0.446, *95% CI* [-0.14, 0.33]. The quadratic term was also not statistically significant and slightly smaller in size compared to the prior baseline, *β* = 0.17, *SE* = 0.11, *p* = 0.135, *95% CI* [-0.05, 0.40]. Nevertheless, this quadratic term effect (*β* = 0.17) lies within the 95% CIs for each of the significant quadratic terms for the first [0.02-0.42] and second [0.09, 0.43] baselines.

**Summary.** The quadratic relation between first-baseline lnHF-HRV and recreational risk-taking was replicated when using cardiac data at the second baseline. Although non-significant (*p* > 0.05), the third-baseline quadratic effect for lnHF-HRV lies within the CIs of the prior baseline effects. With that in mind, the nonlinear relation between lnHF-HRV and recreational risk-taking appears to be somewhat stable across the three baselines, although it may attenuate at the third baseline when recovery effects take hold. Supporting that notion, lnHF-HRV does indeed increase from the second and third baseline (see main Results); stress recovery processes may slightly obscure the resting state “signal” at the last baseline.

**Parsing the Health-Safety DOSPERT Subscale**

Follow-up item-level correlations were conducted on the health/safety subscale, which demonstrated lower consistency, *Cronbach’s alpha* = 0.47. This analysis suggests that the low internal consistency was driven item 1 (“Drinking heavily at a social function”) and item 2 (“Engaging in unprotected sex”) having small negative correlations with some other items, *r*s = -0.07 to -0.19. We therefore computed health-safety risk-taking in two different ways: (1) average score without items 1 and 2, which we term “non-pleasure-seeking” health/safety risk-taking (2) average score of items 1 and 2 only, which we term “pleasure-seeking” health/safety risk-taking. The latter score reflects pleasure-seeking HRB because it assesses binge drinking and risky sexual activity, while the other items assess either a failure to engage in preventative behaviors (items 3 to 5) or walking home at night (item 6).

Regression models were tested on these two separate health/safety scores, allowing us to determine whether focal “full subscale” effects (average across all items; see Results) are driven by particular item sets. Due to positive skew in both variables, both health/safety scores were natural logarithm transformed before running models. Two outliers, determined by Tukeys 1.5*IQR rule, on the non-pleasure-seeking variable was also Winsorized before analysis. The parsed results are detailed below.

**The nonlinear relation between HR and health/safety risk-taking was detected for both “pleasure-seeking” and “non-pleasure-seeking” items.**

We first tested resting HR’s linear and quadratic associations with pleasure-seeking items (binge drinking and risky sex). The linear term for HR was not statistically significant, *β* = 0.04, *SE* = 0.11, *p* = 0.752, *95% CI* [-0.19, 0.26]. However, the positive quadratic term was significant, *β* = 0.21, *SE* = 0.10, *p* = 0.043, *95% CI* [0.01, 0.41].

We next tested HR’s associations with non-pleasure-seeking items. Unlike the linear term, *β* = -0.15, *SE* = 0.10, *p* = 0.136, *95% CI* [-0.34, 0.05] the positive quadratic term was statistically significant, *β* = 0.25, *SE* = 0.12, *p* = 0.029, *95% CI* [0.03, 0.48].

**LnHF-HRV’s nonlinear relation with health/safety risk-taking was only detected for “pleasure-seeking” items.**

We tested resting lnHF-HRV’s linear and quadratic associations with pleasure-seeking items (binge drinking and risky sex). The linear term for lnHF-HRV was not statistically significant, *β* = -0.03, *SE* = 0.11, *p* = 0.760, *95% CI* [-0.25, 0.18]. However, the U-shaped quadratic term was significant, *β* = 0.21, *SE* = 0.10, *p* = 0.039, *95% CI* [0.01, 0.40]. When testing lnHF-HRV’s associations with the “non-pleasure-seeking” items, both the linear term, *β* = 0.18, *SE* = 0.12, *p* = 0.146, *95% CI* [-0.06, 0.42], and quadratic term, *β* = 0.12, *SE* = 0.13, *p* = 0.374, *95% CI* [-0.15, 0.39], for lnHF-HRV were not statistically significant.

**Adjusting for Respiration Rate in HF-HRV Effects**

We sought to address whether respiration rate confounds HF-HRV’s relationships with DOSPERT scores. To do so, respiration rate was first estimated from the low-passed thoracic impedance signal in accordance with published recommendations on impedance pneumography (Ernst et al., 1999). Respiratory intervals were converted to respirate rate values in breath-per-minute units, and mean respiration rate was calculated for each resting baseline. Importantly, due to noisy data affecting all of their baseline periods, four participants were dropped from the analysis (N = 85). If participants had incomplete data, i.e., one missing baseline score, then these scores were imputed with multiple imputation. The three baseline scores were then averaged to arrive at a single baseline score, which was identical to our approach for average lnHF-HRV. The average baseline respiration rate was 16.94 breaths per minute (SD = 1.68). We statistically controlled for average respiration rate as a covariate in the robust regression models testing lnHF-HRV quadratic relations with DOSPERT scores. These models also contained the previously entered covariates: Gender, BMI, time-of-day.

Overall, the regression findings suggest that respiration rate does not account for lnHF-HRV’s quadratic associations with DOSPERT scores (health/safety and recreational). The positive quadratic terms of lnHF-HRV remained statistically significant when adjusting for average respiration rate in both the health/safety, *β* = 0.26, *SE* = 0.11, *p* = 0.015, *95% CI* [0.05, 0.47], and recreational risk-taking, *β* = 0.30, *SE* = 0.10, *p* = 0.004, *95% CI* [0.10, 0.51] models. Since we were focused on quadratic effects of HRV, we additionally controlled for the quadratic term of respiration rate (respiration rate^2^). Again, the quadratic terms of lnHF-HRV remained statistically significant in the health/safety, *β* = 0.26, *SE* = 0.11, *p* = 0.016, *95% CI* [0.05, 0.47], and recreational risk-taking, *β* = 0.33, *SE* = 0.12, *p* = 0.006, *95% CI* [0.10, 0.56], models. The quadratic term for mean HR in the health/safety risk-taking model also remained significant when adjusting for respiration rate’s linear and quadratic terms, *β* = 0.23, *SE* = 0.09, *p* = 0.017, *95% CI* [0.04, 0.42].

**
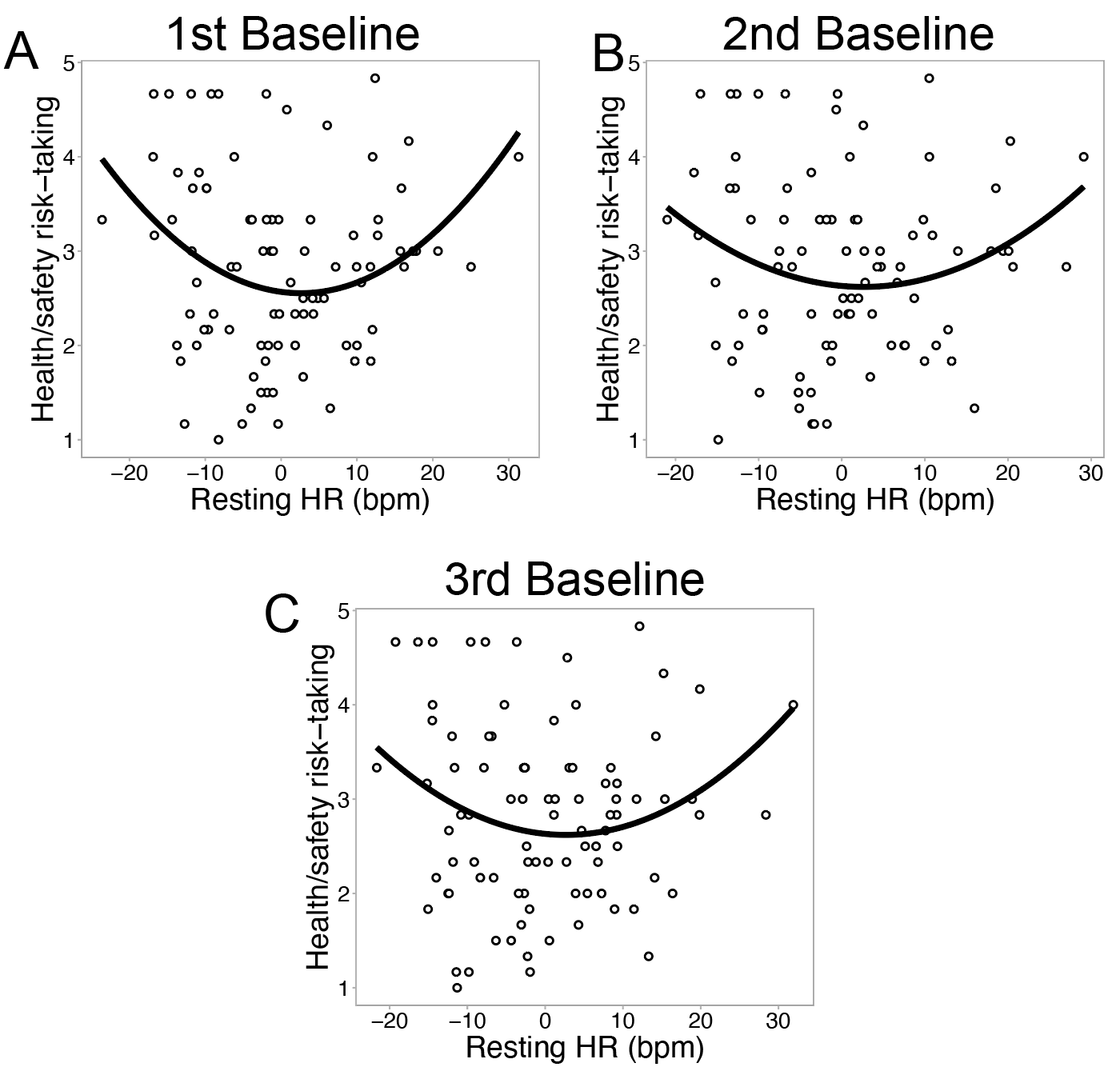
**

**Figure S1.** Quadratic Associations between Resting HR and DOSPERT Risk-Taking Propensity in the Health/Safety Domain. Each panel (A-C) corresponds to a different baseline period, from which resting HR was computed.

**
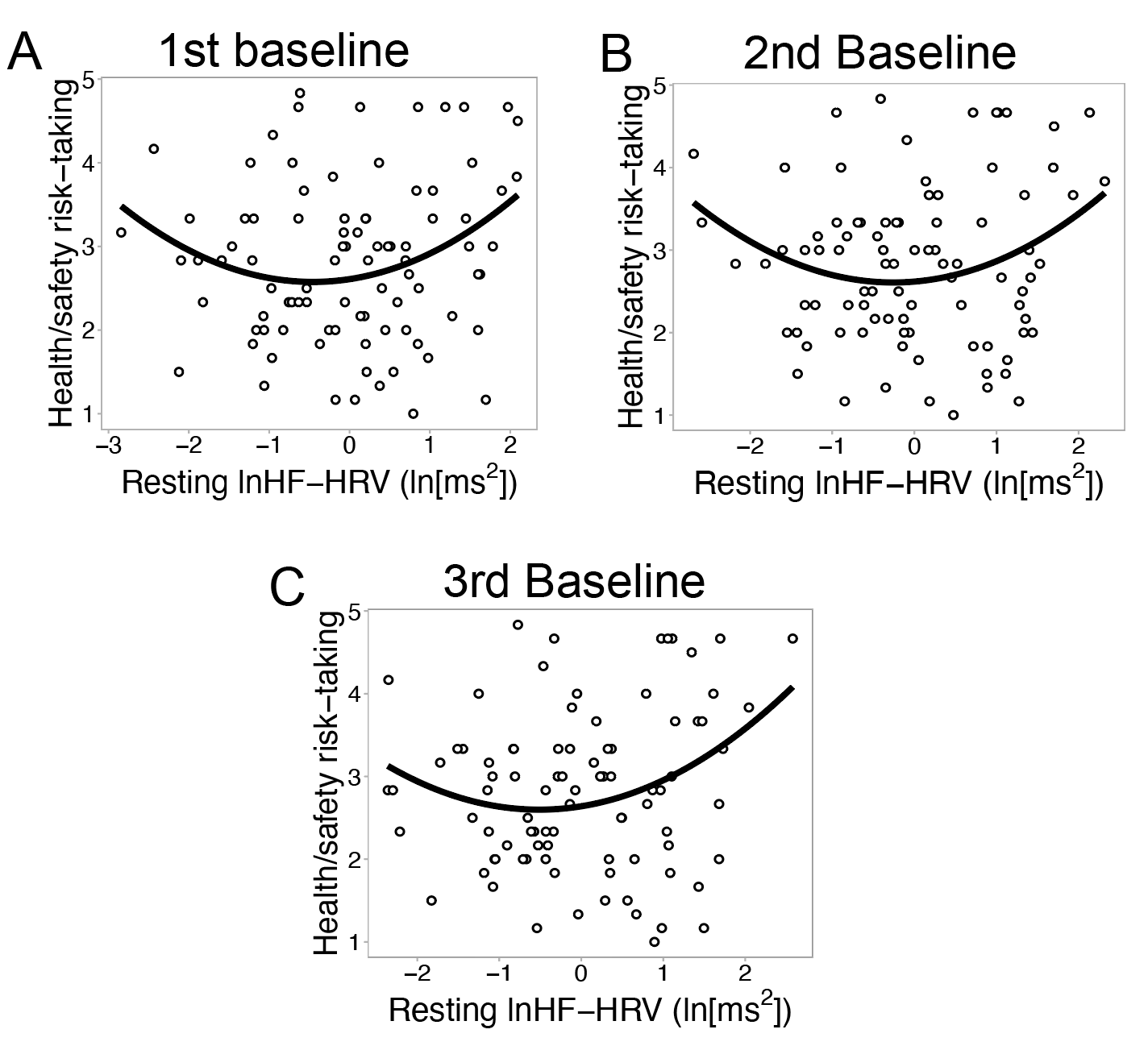
**

**Figure S2.** Quadratic Associations between Resting lnHF-HRV and DOSPERT Risk-Taking Propensity in the Health/Safety Domain. Each panel (A-C) corresponds to a different baseline period, from which resting lnHF-HRV was computed.

**
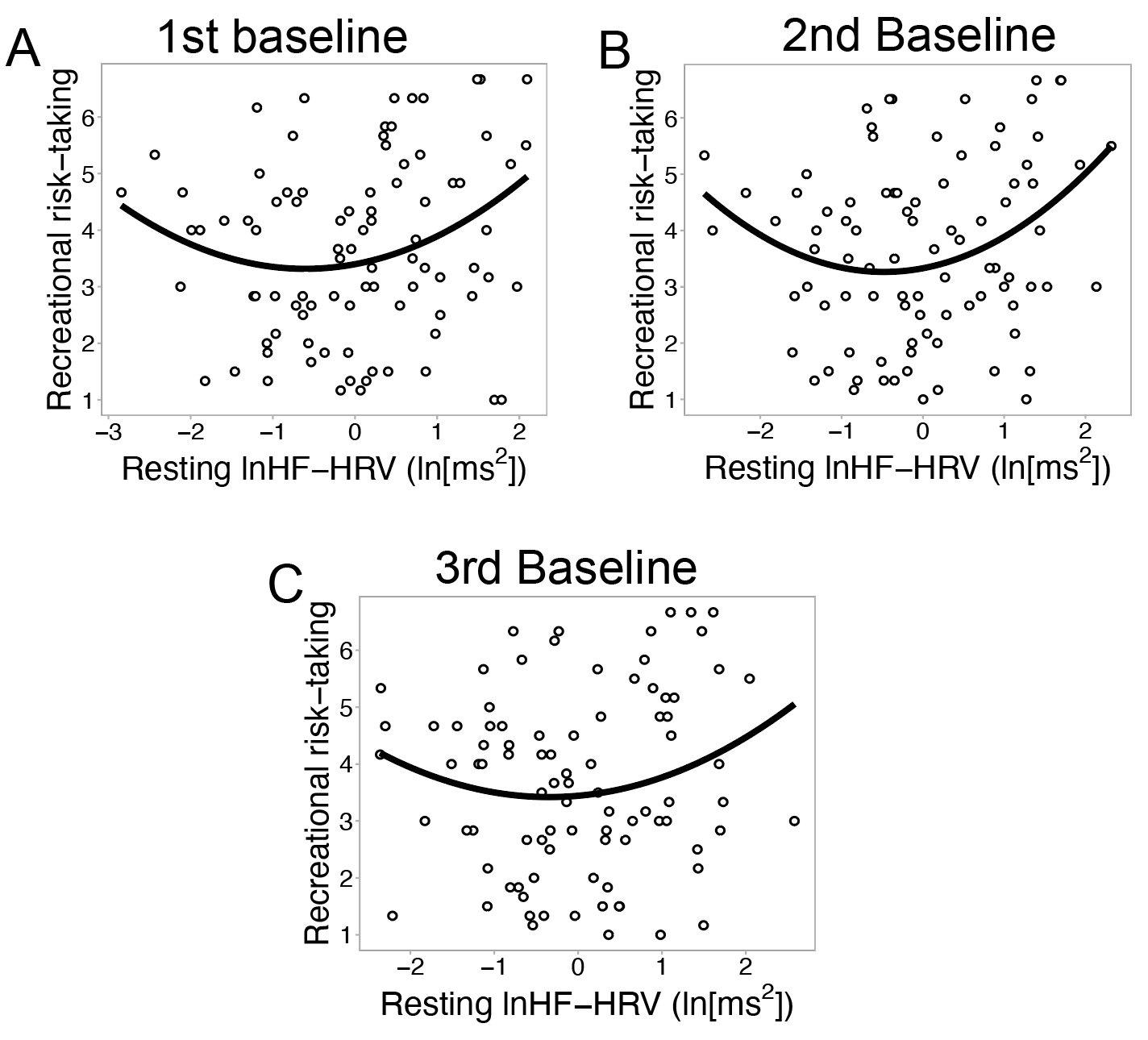
**

**Figure S3.** Quadratic Associations between Resting lnHF-HRV and DOSPERT Risk-Taking Propensity in the Recreational Domain. Each panel (A-C) corresponds to a different baseline period, from which resting lnHF-HRV was computed.
